# 805 Peer Support and Long-Term Community Integration: Enhancing Recovery for Burn Survivors

**DOI:** 10.1093/jbcr/iraf019.336

**Published:** 2025-04-01

**Authors:** Dania Johnson, Kara McMullen, Elizabeth Flores, Caitlin Orton, Jennifer Bell-De Paz, Jill Sproul, Cindy Rutter, Haig Yenikomshian

**Affiliations:** University of California Keck School of Medicine; University of Washington; University of Southern California / LA General Burn Unit; University of Washington; University of Texas Southwestern; Phoenix Society for Burn Survivors; Southern California Burn Model System; University of California Keck School of Medicine

## Abstract

**Introduction:**

Peer support fosters community and encouragement among burn survivors through shared experiences. Despite its growing recognition, the impact on patient-reported outcomes is rarely studied. This study explores the effects of peer support on psychosocial and functional outcomes.

**Methods:**

This retrospective cross-sectional study analyzed data from participants over the age of 18 from 2014-2024 in a multicenter longitudinal patient-reported outcome database. Participant demographics, PROMIS domains (ability to participate in social roles, anxiety, depression, sexual satisfaction), community integration – social integration (CIQ), and satisfaction with life (SWL) were collected at 12 months post-injury. Peer support was assessed at 12 months by asking participants if they had spoken with other burn survivors for support regarding their burn injuries since the last questionnaire. Univariate analyses compared outcomes based on peer support. Wilcoxon-Mann-Whitney tests were used due to non-parametric nature of the data. Six linear regression models evaluated the impact of peer support (independent variable) on 12-month outcomes (depression, anxiety, sexual satisfaction, CIQ, SWL, and social role participation), adjusting for age, sex, TBSA, burn center site, and education level. Each model included the outcome measure not being assessed to control for the impact of those domains on the dependent variable.

**Results:**

Data from 700 participants met inclusion criteria with 106 receiving peer support and 594 not. At 12 months, peer support was associated with significantly lower scores for PROMIS ability to participate in social roles (50.4+/- 10.9 vs. 54.0+/- 10.5, p=0.0007), anxiety (52.4 +/- 9.8 vs. 49.4 +/- 10.2, p=0.0018), depression (52.2 +/- 11.3 vs. 48.6 +/- 9.8, 0.0014), and satisfaction with life (16.7 +/- 7.1 vs. 19.2 +/- 7.0, p=0.0009) compared to those not receiving peer support. Linear regression results showed that, when adjusted for age, sex, TBSA, burn center site, and more than a high school education, peer support was not significantly associated with ability to participate in social roles, anxiety, depression, sexual satisfaction, satisfaction with life, or community integration.

**Conclusions:**

Individuals seeking peer support had worse patient-reported outcomes, likely due to more severe injuries as outcomes were not different between the two groups when adjusted for burn size. The above data helps justify the need for peer support programs as routine care for burn survivors to help in proper recovery.

**Applicability of Research to Practice:**

This study highlights the need to integrate peer support programs into standard practice for burn survivors, emphasizing training for volunteers to discuss these concerns.

**Funding for the Study:**

The contents of this abstract were developed under a grant from the National Institute on Disability, Independent Living, and Rehabilitation Research (NIDILRR grant number 90DPBU0007). NIDILRR is a Center within the Administration for Community Living (ACL), Department of health and Human Services (HHS). The contents of this abstract do not necessarily represent the policy of NIDILRR, ACL, or HHS, and you should not assume endorsement by the Federal Government.

